# Osteoblastogenesis from synovial fluid-derived cells is related to the type and severity of juvenile idiopathic arthritis

**DOI:** 10.1186/ar3872

**Published:** 2012-06-12

**Authors:** Elvira Lazić, Marija Jelušić, Danka Grčević, Ana Marušić, Nataša Kovačić

**Affiliations:** 1 Laboratory for Molecular Immunology, University of Zagreb School of Medicine, Salata 12, Zagreb-HR 10000, Croatia

## Abstract

**Introduction:**

Juvenile idiopathic arthritis (JIA) is characterized by synovial inflammation, followed by hyperplastic changes of the synovium, and destruction of articular cartilage along with underlying bone. This hyperplastic process is the result of inflammation-induced activation of NF-κB, which may be accompanied by decreased osteogenic differentiation of synovial mesenchymal progenitors and contribute to bone resorption. We aimed to explore osteoblast differentiation of synovial fluid (SF)-derived mesenchymal progenitors and correlate it with intensity of inflammation in patients with JIA.

**Methods:**

Peripheral blood from 18 patients with oligoarticular (o)JIA, 22 patients with polyarticular (p)JIA and 18 controls was collected along with SF from 18 patients with oJIA and 9 patients with pJIA. SF-derived cells were cultured to assess osteoblastogenesis, using alkaline phosphatase histochemical staining and colorimetric activity assay. The expression of osteoblast-related genes, Runt-related transcription factor 2 (*Runx2*), Osteoprotegerin (*OPG*), Receptor activator of nuclear factor κB ligand (*RANKL*) and arthritis-related cytokine/chemokine genes, Tumor necrosis factor alpha (*TNF-α*, *Fas*, Fas ligand (*FasL*), Interleukin (*IL)-1β*, *IL-4*, *IL-6*, *IL-17*, *IL-18*, CC chemokine ligand (*CCL)-2*, *CCL3*, *CCL4 *was evaluated. Osteoblastogenesis was correlated with systemic and local inflammatory indicators. Expression of osteoblast genes was also analyzed in peripheral blood mononuclear cells (PBMC) and total SF-derived cells from patients with JIA. Additionally, we assessed the inhibitory effect of SF from patients with JIA on differentiation of human bone marrow (hBM)-derived osteoblasts.

**Results:**

Osteoblastogenesis from SF-derived progenitors was decreased in patients with pJIA compared to those with oJIA. Osteoblastogenesis from primary SF-derived cells negatively correlated with erythrocyte sedimentation rate (ρ = -0.391, *P *= 0.05), C-reactive protein concentration (ρ = -0.527, *P*<0.01) and synovial concentration of IL-17 (ρ = -0.552, *P *= 0.01). SF-derived osteoblasts from pJIA patients expressed more *CCL2 *and *CCL3 *genes than in oJIA (*P *= 0.04 and *P *= 0.03, respectively; Mann-Whitney test). Expression of *Fas *was significantly higher in osteoblasts from patients with pJIA than those with oJIA (*P *= 0.03, Mann-Whitney test). SF-derived cells from patients with pJIA expressed higher levels of *RANKL *than in oJIA (*P *= 0.05, Mann-Whitney test). PBMCs from patients with JIA expressed less *OPG *than healthy control patients (*P *= 0.05, Kruskal-Wallis test). SF from all tested JIA patients inhibited differentiation of hBM-derived osteoblasts (*P *= 0.04, Kruskal-Wallis test).

**Conclusions:**

Osteoblast differentiation was decreased in patients with severe forms of JIA and accompanied by altered cytokine/chemokine expression pattern. Development of therapeutic interventions targeting synovial mesenchymal or osteoblast lineage cells in JIA would contribute to alleviating both bone destruction and inflammation in severe forms of the disease.

## Introduction

Juvenile idiopathic arthritis (JIA) is one of the leading causes of disability in children, characterized by synovial hyperplasia and formation of pannus, which cause destruction of articular cartilage and underlying bone [1,2]. Clinically, JIA is defined as arthritis appearing before 16 years of age, with a minimum duration of six or more weeks and exclusion of other forms of childhood arthritis [3]. According to the International League of Associations for Rheumatology (ILAR) JIA is classified into the following subtypes: systemic JIA, oligoarticular JIA (oJIA, affecting four or fewer joints), polyarticular JIA (pJIA, affecting five or more joints), psoriatic arthritis, enthesitis-related arthritis and undifferentiated arthritis [3]. In general, oJIA is the most frequent disease form (56 to 60% of cases), followed by pJIA (28 to 30%). The course of disease is variable; patients with oJIA have the best outcome, while the course of pJIA is characterised by progressive and diffuse joint involvement and early radiographic changes.

The pathogenesis of bone loss in children with JIA involves inflammation, physical inactivity, medication intake and malnutrition [4]. Inflammation-induced bone loss in JIA is driven by the interactions of the immune system and bone, which share a number of regulatory molecules [5]. The stimulatory effects of inflammation on osteoclast-mediated bone resorption are well established but the influence of pro-inflammatory cytokines on osteoblast function *in vivo *requires further elucidation [6]. It is known that tumor necrosis factor (TNF) and interleukin (IL)-1β may directly impair osteoblast differentiation [7-10]. Bone marrow-derived mesenchymal stem cells (MSC) from TNF transgenic mice, that develop chronic inflammatory arthritis, form fewer osteoblast colonies with decreased expression of osteoblast genes [11]. In addition, pro-inflammatory factors are able to disrupt the Wnt signaling pathway, which normally induces the differentiation and maturation of osteoblasts [12,13]. Among Wnt antagonists, murine models revealed that Dickkopf and secreted Frizzled-related proteins are upregulated in arthritic synovial tissues and may contribute to decreased osteoblast function [14]. Inhibition of Dickkopf-1 was able to reverse bone destruction towards bone formation in the mouse model of rheumatoid arthritis (RA) [12]. The involvement of Fas and Fas ligand (FasL) has also been proposed in osteoblast differentiation, and confirmed in animal models of Fas deficiency, which is found to protect animals from osteoporosis and joint destruction in arthritis [15-17].

Therapeutic treatment of joint destruction in JIA aims to attenuate inflammation and bone resorption, as well as to increase regeneration of subchondral bone and cartilage. Several therapeutic approaches involve MSC because of their immunomodulatory and regenerative capacity [18]. MSC are multipotent cells with ability to differentiate into osteoblasts, chondroblasts, adipocytes, connective tissue fibroblasts and myoblasts, thus contributing to tissue regeneration [18]. They are present in various tissues such as bone marrow, skin, adipose and connective tissues, as well as the synovia [19,20].

In a murine model, synovial hyperplasia develops as a result of inflammation-induced activation of nuclear factor κB (NF-κB) in synovial fibroblasts, which promotes their proliferation and inhibits osteoblast/chondroblast lineage differentiation [21,22]. This mechanism could also be relevant for human JIA, promoting inflammation and suppressing joint regeneration.

The overall aim of the present study was to assess the osteoblastogenesis from synovial fluid (SF)-derived cells in patients with JIA and its association with disease type and activity. We first assessed osteoblastogenesis and osteoblast gene expression in SF-derived cells from patients with JIA and correlated them with systemic and local inflammatory activity. We also assessed the systemic expression of osteoblast-related genes in patients with JIA and control patients, as well as cytokine and chemokine expression in osteoblasts from JIA patients. Finally, we investigated the effect of SF from patients with JIA on the differentiation of human bone marrow (hBM)-derived osteoblasts.

## Materials and methods

### Patients

A total of 40 children (29 girls and 11 boys, Table 1), admitted to the Division of Paediatric Rheumatology of University Hospital Centre Zagreb, between December 2008 and December 2010 with the diagnosis of JIA, were included in the study after obtaining approval from the regional Ethics Committee and informed consent from patients. oJIA was diagnosed in 18 and pJIA in 22 children, in accordance with the ILAR criteria [3]. Eight patients with oJIA and ten patients with pJIA did not receive any therapy at the time of sampling, and the others received non-steroid anti-inflammatory drugs (NSAID) (5 patients each with oJIA and pJIA); disease modifying anti-rheumatic drugs (DMARDs) - methotrexate (MTX) (5 oJIA, 4 pJIA); anti-TNF - etanercept (1 pJIA) or MTX + prednisone (2 pJIA).

**Table 1:** Demographic and laboratory data for juvenile idiopathic arthritis (JIA) patients and controls*

Characteristic	Control (n = 18)	oJIA (n = 18)	pJIA (n = 22)
Age, years	7.33 ± 5.22	7.13 ± 5.20	11.36 ± 4.50
Male/Female	6/12	4/14	7/15
ESR (1 h)	8.33 ± 4.27	16.44 ± 9.46	53.09 ± 41.03
CRP (g/L)	4.39 ± 6.80	4.34 ± 6.36	47.31 ± 46.3
Leukocyte number (x10^9^/L)	6.54 ± 1.65	10.03 ± 4.01	9.57 ± 4.62
Hb (g/L)	124 ± 9.70	123.69 ± 11.36	117.4 ± 14.02

*Values are mean ± standard deviation. Patients were subdivided into oligoarticular (oJIA) or polyarticular (pJIA) group according to the International League of Associations for Rheumatology (ILAR) criteria [3]. ESR, erythrocyte sedimentation rate; CRP, C-reactive protein; Hb, hemoglobin.

Disease activity was followed by clinical examination and laboratory assessment (Table 1).

Healthy children without history of autoimmune or joint diseases, admitted to the Division due to non-inflammatory conditions during the same period were also included in the study as controls (n = 18, 12 girls and 6 boys, Table 1) after obtaining informed consent.

Peripheral blood was obtained from patients and controls during routine clinical assessment, followed by peripheral blood mononuclear cell (PBMC) separation using Histopaque (Sigma-Aldrich, St. Louis, MO, USA). All participants' samples were obtained in the morning after overnight fasting. In addition, SF samples were collected at the same time from children with JIA with indication for arthrocentesis (18 patients with oJIA and 9 patients with pJIA), and synovial cells were separated by centrifuging. Serum and SF samples were collected in aliquots of 500 μL and stored at -20°C until used. Routine laboratory tests were performed at the Department of Clinical Laboratory Diagnostics of the same Hospital Center.

### Osteoblast differentiation from synovial fluid-derived cells

SF-derived cells were cultured in a density of 0.75 × 10^6 ^cells/cm^2 ^in 24-well plates in minimal essential medium-α (α MEM) supplemented with 10% fetal bovine serum (FBS). Osteoblast differentiation was induced on culture day 7 by the addition of 50 μg/mL ascorbic acid and 5 mM β-glycerophosphate, and assessed on culture day 21 by alkaline phosphatase (AP) histochemical staining using a commercially available kit (Sigma Aldrich, Milwaukee, MI, USA). Since colonies formed by SF-derived cells were poorly delineated and confluent, the area with the red staining was measured by image-analyzing custom made software for quantification of osteoblast differentiation. Total cellularity in each well was estimated by staining with methylene blue (MB), and quantified as the area of blue stain using the same software. Osteoblast differentiation was additionally assessed by measuring expression of osteoblast-specific genes on culture days 14 and 21 by real-time polymerase chain reaction (PCR). To expand SF-derived mesenchymal progenitors and remove inflammatory and hematopoietic cells from the culture, adherent cells were passaged three times, and osteoblastogenesis was induced only in the fourth passage (P4) cells by plating 0.5 × 10^5 ^cells/cm^2 ^in α-MEM/10% FBS in 24-well plates and addition of 50 μg/mL ascorbic acid and 5 mM β-glycerophosphate on culture day 2. Assessment of osteoblast differentiation was performed on day 14 by AP histochemical staining using a commercially available kit (Sigma Aldrich), and expression of osteoblast genes on culture days 10 and 14 by real-time PCR. Total cellularity in each well was estimated by staining with MB. Osteoblast differentiation was quantified by AP activity colorimetric assay (Sigma Aldrich), reflecting the number of active osteoblasts per well.

The time points for gene expression analysis were determined in a preliminary set of experiments by multiple time point analysis of expression of differentiation genes in human primary and P4 SF-derived osteoblasts. Based on these data, we chose optimal time points which reflected immature and mature stage of osteoblast differentiation.

### Bone marrow osteoblast culture

Normal hBM was obtained from a healthy donor after obtaining approval from the regional Ethics Committee and informed consent from the patient. The 2 × 10^6 ^cells were plated in 75 cm^2 ^flasks and cultured in α-MEM medium supplemented with 10% FBS until reaching confluence. After two passages, 5 × 10^3 ^cells/cm^2 ^were plated in a 24-well culture plate. After reaching confluence, control cells were grown in α-MEM/10% FBS, 50 μg/mL ascorbic acid and 5 mM β-glycerophosphate for 17 days. Additionally, cells were cultured with 10% SF from oJIA or pJIA patients. Osteoblastogenesis was assessed by AP activity colorimetric assay (Sigma Aldrich) and expression of osteoblast genes by real-time PCR.

### Alkaline phosphatase colorimetric assay

For the AP activity assay, total cells from two wells plated in a density of 0.75 × 10^6 ^cells/cm^2 ^for primary cultures, and 0.5 × 10^5 ^cells/cm^2 ^for P4 cultures, were pooled for analysis. Upon removal of cell culture media, cells were washed 3 times with phosphate-buffered saline (PBS) and lysed with 200 μL lysing buffer (10 mM Tris, 0.1% Triton X-100, pH 7.5) per well. AP activity was measured in a 96-well plate. Five μL of the sample and 15 μL of lysing buffer were added in duplicates into each well, together with 180 μL p-nitrophenyl phosphate (pNP) solution (Sigma Aldrich) and incubated for 30 minutes at 37°C. Color development was measured at 405 nm using the enzyme-linked immunosorbent assay (ELISA) microplate reader (Bio-Rad, Hercules, CA, USA). Results were expressed as micromoles of pNP per hour.

### Gene expression

Total RNA was extracted from peripheral blood cells and SF-derived cells using 6100 Nucleic Acid PrepStation (Applied Biosystems, Foster City, CA, USA). For PCR amplification, 1 μg of total RNA was converted to complementary DNA (cDNA) by reverse transcriptase (Applied Biosystems). The amount of cDNA corresponding to 20 ng of reversely transcribed RNA was amplified by real-time PCR. Expression of glyceraldehyde 3-phosphate dehydrogenase (*GAPDH*), runt-related transcription factor 2 (*Runx2*), osteoprotegerin (*OPG*), receptor activator of NF-κB ligand (*RANKL*), *TNF-α*, *Fas*, *Fas ligand*, *IL-1β*, *IL-4*, *IL-6*, *IL-17*, *IL-18*, CC chemokine ligand (*CCL) 2*, *CCL3*, and *CCL4 *was analyzed using commercially available TaqMan Assays (Applied Biosystems).

Real-time PCR was conducted using the ABI Prism 7500 Sequence Detection System (Applied Biosystems). Each reaction was performed in duplicate in a 25 μL reaction volume. The relative quantities were calculated using the standard curve designed from 6 serial dilutions of the calibrator sample (control PBMC, SF-derived cells or osteoblast culture cells). According to the standard curve, the relative amounts of messenger RNA for target genes were calculated as the ratio of the quantity of the target gene normalized to *GAPDH *as the endogenous control.

### ELISA

The concentration of IL-17 and TNF-α in SF was determined using a commercial kit (Quantikine, Human IL-17 Immunoassay and Quantikine High Sensitivity, Human TNF-α Immunoassay, R&D systems, Minneapolis, MN, USA). Briefly, samples were added to anti-IL-17 or anti-TNF-α monoclonal antibody-precoated plates and incubated for 2 or 3 hours respectively at room temperature, washed five times and incubated for the next 2 hours with horseradish peroxidase conjugated IL-17 or AP-conjugated TNF-α-specific Ab. After five further washes, the reaction was visualized with substrate solution (tetramethylbenzidine) for IL-17 or substrate and amplifier solutions (NADPH and amplifier enzymes) for TNF-α, and arrested with hydrochloric or sulfuric acid respectively. Optical density was determined within 15 minutes, on the microplate reader (Bio-Rad, Hercules, CA, USA) set to the excitation wavelength at 450 nm or 490 nm respectively.

### Statistical analysis

Clinical and laboratory data for each type of arthritis were presented as mean ± standard deviation (SD) and compared using analysis of variance. AP activity, AP staining intensity, and gene expression values in JIA and control samples were expressed as median with interquartile range (IQR) and compared using the nonparametric Kruskal-Wallis test followed by the Mann-Whitney test and Bonferroni's correction for multiple testing. Osteoblast differentiation parameters were additionally correlated to inflammation markers using rank correlation and Spearman's coefficient rho (ρ) with its 95% confidence interval (CI). Statistical analysis was performed using the MedCalc software package (Mariakerke, Belgium). For all experiments, the α-level was set at 0.05.

## Results

### Osteoblast differentiation from synovial fluid-derived cells in patients with JIA

**Figure 1:**
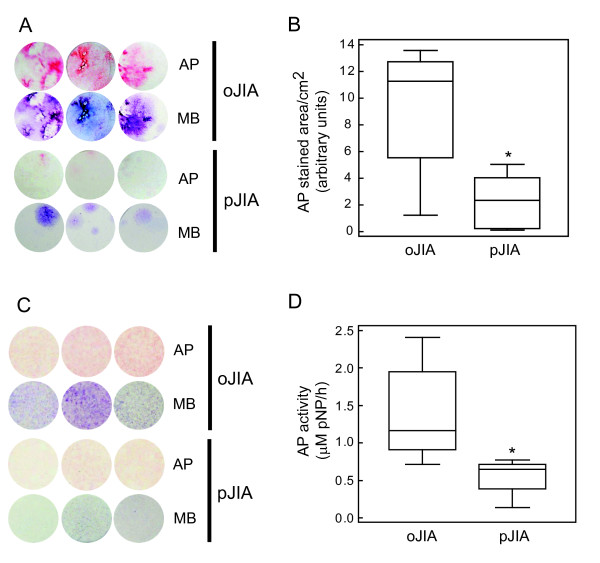
**Differentiation of osteoblasts from primary synovial fluid (SF)-derived cells and SF-derived fibroblasts from the fourth passage (P4), from patients with oligoarticular juvenile idiopathic arthritis (oJIA) and polyarticular JIA (pJIA)**. Osteoblast differentiation was induced by ascorbic acid and β-glycerophosphate, and assessed by alkaline phosphatase (AP) histochemical staining. Total fibroblastic colonies were visualized with methylene blue (MB). **(A) **Osteoblast and total colonies from representative primary SF-derived cells from children with oJIA and pJIA stained for AP and MB on culture day 21. **(B) **AP-stained area (median and interquartile range (IQR), lines represent minimum and maximum values) in osteoblast cultures grown from primary SF-derived cells from children with oJIA (n = 18) and pJIA (n = 9); * *P *< 0.05 vs. oJIA group, Mann-Whitney test. **(C) **Osteoblast and total colonies from representative SF-derived fibroblast samples from the fourth passage (P4) of SF-derived cells from children with oJIA (n = 7) and pJIA (n = 4) stained for AP and MB on culture day 14. **(D) **AP activity measured at day 14 in osteoblast cultures derived from P4 SF-derived fibroblast population (median and IQR);* *P *< 0.05 vs. oJIA, Mann-Whitney test).

SF-derived cells from oJIA formed more AP-positive, osteoblast-like colonies than SF-derived cells from patients with pJIA when plated at the same density (Figure 1A, B). At the same time, total cellularity assessed by MB staining was also higher in cultures of SF-derived cells from patients with oJIA in comparison to those with pJIA, (median 11.49, IQR 8.23 to 12.82 vs. median 5.23, IQR 0.43 to 6.47, arbitrary units/cm^2^, *P *= 0.004, Mann-Whitney test). Similar findings were observed in osteoblastogenic cultures from adherent SF-derived cells after removal of inflammatory cells by cell passaging (P4-cells). As the difference in the histochemical staining intensity was not so prominent in P4 cultures, since P4-cells grew in homogenous monolayers (Figure 1C), we used AP activity colorimetric assay to quantitatively assess osteoblast differentiation. AP activity was significantly higher in osteoblastogenic cultures from patients with oJIA, than in those with pJIA (Figure 1D). When P4-cells from patients with oJIA were plated at equal densities, total cellularity (MB staining, Figure 1C) was higher than in patients with pJIA (median 11.09, IQR 6.52 to 12.10 vs. median 2.41, IQR 1.74 to 5.91, arbitrary units/cm^2^, *P *= 0.04, Mann-Whitney test).

In addition, the second passage was efficient only in 4 out of 9 pJIA patients, significantly less in comparison to oJIA patients (16 out of 18, Fisher's exact test, *P *= 0.0235).

**Figure 2:**
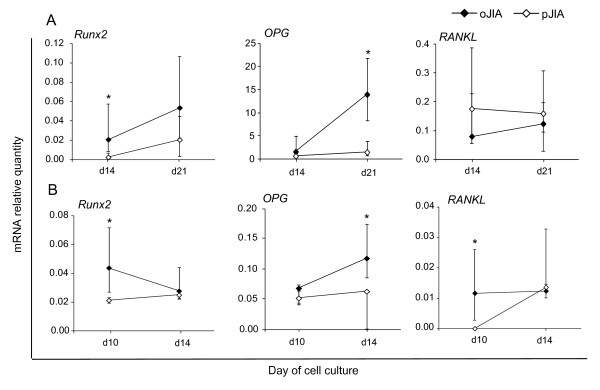
**Real time PCR analysis of expression of early (*Runx2*) and late (*OPG, RANKL*) osteoblast genes in synovial fluid (SF)-derived osteoblasts**. **(A)** Primary SF-derived osteoblastogenic cultures from children with oligoarticular form of juvenile idiopathic arthritis (oJIA, n = 12) and polyarticular form of JIA (pJIA, n = 4) at culture days 14 and 21 (median and interquartile range (IQR), * *P *< 0.05 vs. pJIA, Mann-Whitney test). **(B) **Osteoblastogenic cultures from the fourth passage (P4) of SF-derived cells from children with oJIA (n = 7) and pJIA (n = 4), at culture days 10 and 14 (median and IQR,* *P *< 0.05 vs. pJIA, Mann-Whitney test). mRNA expression was calculated according to the standard curve for each gene expression in the calibrator sample (cDNA from osteoblast cultures) and normalized to the mRNA quantity for glyceraldehyde 3-phosphate dehydrogenase (*GAPDH*) (endogenous control). *OPG*, osteoprotegerin; *RANKL*, receptor activator of NF-κB ligand; *Runx2*, runt-related transcription factor 2.

Inhibition of osteoblast differentiation was confirmed at the level of osteoblast gene expression as the early osteoblast marker *Runx2*, essential for osteoblast differentiation [23], was significantly decreased in pJIA on day 14 of the primary SF-derived osteoblast culture (*P *= 0.02, Mann-Whitney test; Figure 2A, Table 2), and day 10 of P4-SF-derived cell culture (*P *= 0.02, Mann-Whitney test; Figure 2B). *OPG *gene expression, the marker of mature osteoblasts, was significantly decreased in pJIA on day 21 of the primary SF-derived osteoblast culture (*P *= 0.03, Mann-Whitney test; Figure 2A, Table 2), and day 14 of the P4-SF-derived cell culture (*P *= 0.01, Mann-Whitney test; Figure 2B, Table 2). Expression of *RANKL *was significantly lower in pJIA only on culture day 10 in P4-SF-derived osteoblast culture (*P *= 0.04 Mann-Whitney test; Figure 2B, Table 2).

**Table 2:** Osteoblast gene expression in primary and fourth passage (P4) SF-derived osteoblast cultures from patients with juvenile idiopathic arthritis*

	Primary cultures			
	
Gene	oJIA (n = 12)		pJIA (n = 4)	
	
	Day 14	Day 21	Day 14	Day 21
*Runx2*	0.02 (0.01-0.07)	0.05 (0.02-0.11)	0.00 (0.00-0.01)	0.02 (0.00-0.05)
*P *(vs. pJIA)	0.02	0.18		
*P *(vs. day 21)	0.20		0.34	
*OPG*	1.63 (0.39-5.65)	13.99 (8.22-21.82)	0.68 (0.40-1.48)	1.52 (0.50-5.68)
*P *(vs. pJIA)	0.47	0.03		
*P *(vs. day 21)	0.001		0.49	
*RANKL*	0.08 (0.00-0.16)	0.18 (0.04-0.50)	0.12 (0.03-0.20)	0.16 (0.06-0.20)
*P *(vs. pJIA)	0.54	0.90		
*P *(vs. day 21)	0.42		0.89	

	**P4 cultures**			
	
	**oJIA (n = 7)**		**pJIA (n = 4)**	
	
	**Day 10**	**Day 14**	**Day 10**	**Day 14**
	
*Runx2*	0.04 (0.02-0.08)	0.03 (0.02-0.05)	0.02 (0.01-0.02)	0.02 (0.01-0.03)
*P *(vs. pJIA)	0.02	0.32		
*P *(vs. day14)	0.46		0.20	
*OPG*	0.07 (0.04-0.08)	0.12 (0.08-0.20)	0.04 (0.01-0.06)	0.04 (0.01-0.06)
*P *(vs. pJIA)	0.23	0.01		
*P *(vs. day14)	0.04		0.89	
*RANKL*	0.01 (0.01-0.03)	0.01 (0.01-0.04)	0.00 (0.00-0.00)	0.01 (0.00-0.01)
*P *(vs. pJIA)	0.04	0.23		
*P *(vs. day14)	0.53		0.06	

*Values are median (interquartile range), statistical differences were calculated using the Mann-Whitney test. Patients were subdivided into the oligoarticular (oJIA) or polyarticular (pJIA) group according to the International League of Associations for Rheumatology (ILAR) criteria [3]. Day, day of osteoblast culture; *OPG*, osteoprotegerin gene; *RANKL*, receptor activator of NF-κB ligand gene; *Runx2*, runt-related transcription factor 2 gene.

### Expression of osteoblast genes in synovial fluid-derived cells and peripheral blood mononuclear cells from patients with JIA

**Figure 3:**
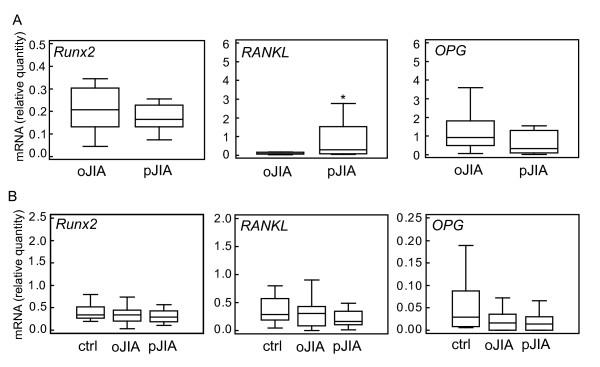
**Real time PCR analysis of expression of osteoblast genes in synovial fluid (SF)-derived cells (A) and peripheral blood mononuclear cells (PBMC) from patients with oligoarticular form of juvenile idiopathic arthritis (oJIA), polyarticular form of JIA (pJIA), and control (ctrl) children**. **(B) **mRNA expression was calculated according to the standard curve for each gene expression in the calibrator sample (cDNA from SF-derived cells or PBMC) and normalized to mRNA quantity for glyceraldehyde 3-phosphate dehydrogenase (*GAPDH*) (endogenous control). Results are presented as median and interquartile range (IQR) for each group; lines represent minimum and maximum values; * *P *≤ 0.05 vs. oJIA (Mann-Whitney test). PBMC, peripheral blood mononuclear cell; *OPG*, osteoprotegerin; *RANKL*, receptor activator of NF-κB ligand; *Runx2*, runt-related transcription factor 2.

Primary SF-derived cells from patients with oJIA and pJIA had similar expression of the early osteoblast marker *Runx2*, as well as the late osteoblast marker *OPG *gene (Figure 3A). Expression of *RANKL *was significantly higher in patients with pJIA than in patients with oJIA (*P *= 0.05, Mann-Whitney test; Figure 3A). PBMCs from patients with JIA expressed less *OPG *than healthy control patients (*P *= 0.05, Kruskal-Wallis test: Figure 3B), whereas there was no difference in the expression of *RANKL *and *Runx2 *between the patient and control groups (Figure 3B).

### Osteoblast differentiation negatively correlated with systemic and local inflammatory indicators

**Figure 4:**
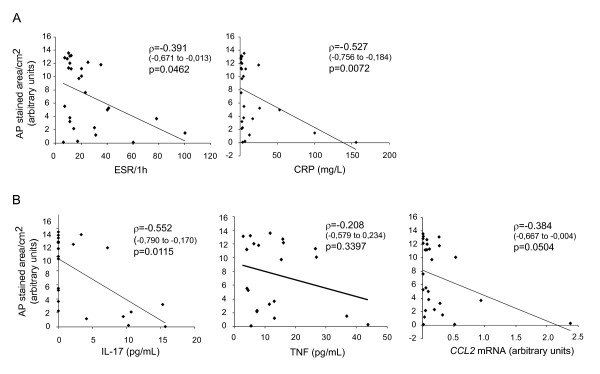
**Correlation of alkaline phosphatase (AP) staining with inflammatory parameters**. **(A) **Systemic inflammatory parameters: erythrocyte sedimentation rate (ESR) and C reactive protein (CRP). **(B) **Local inflammatory parameters: synovial concentration of interleukin (IL)-17 and TNF-α; and *CCL2 *mRNA expression in synovial fluid (SF)-derived cells. Values were correlated using rank correlation and Spearman's coefficient ρ (95% confidence interval for ρ).

As estimated by the area of the red stain of AP-histochemically positive colonies, SF-derived osteoblast differentiation negatively correlated with erythrocyte sedimentation rate (ESR, *P *= 0.05, rank correlation; Figure 4A). The correlation was stronger for C-reactive protein (CRP, *P *< 0.01, rank correlation; Figure 4A). Furthermore, osteoblast differentiation negatively correlated with local synovial concentration of IL-17 (*P *= 0.01, rank correlation; Figure 4B), an inflammatory cytokine closely related to the development of bone erosions and produced by Th17 cells, which are enriched in joints of children with arthritis [24,25]. The inverse relation was also found between synovial concentrations of TNF-α and osteoblastogenesis from SF-derived progenitors, although the correlation was not statistically significant (*P *= 0.34, rank correlation; Figure 4B). Finally, osteoblast differentiation negatively correlated with expression of the *CCL2 *gene in SF-derived cells (*P *= 0.05, rank correlation; Figure 4B).

### Expression of cytokines and chemokines in osteoblasts from patients with JIA

Mesenchymal lineage cells, as well as differentiating and mature osteoblasts, are known to have immunoregulatory properties, expressing various pro- or anti-inflammatory cytokines and chemokines [26]. We assessed gene expression of several candidate cytokines and chemokines which could be involved in the regulation of inflammation in the course of JIA.

**Figure 5:**
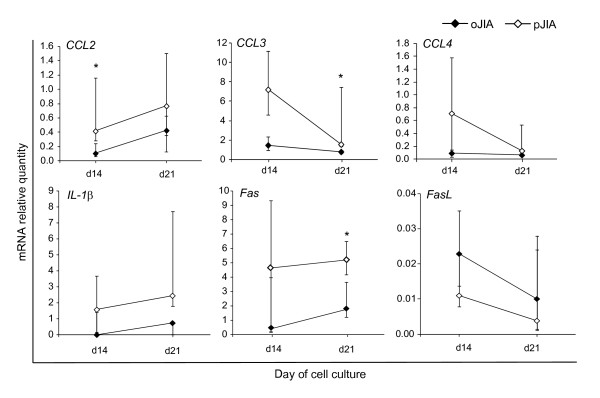
**Expression of chemokines *CCL2*, *CCL3*, *CCL4*, and *IL-1β*, *Fas *and *Fas ligand *(*FasL*) genes in primary synovial fluid (SF)-derived osteoblastogenic cultures**. Cultures were derived from children with oligoarticular juvenile idiopathic arthritis (oJIA, n = 12) or polyarticular JIA (pJIA, n = 4) and gene expression analyzed at culture days 14 and 21 (median and interquartile range (IQR), *P < 0.05 vs. oJIA, Mann-Whitney test). mRNA expression was calculated according to the standard curve for each gene expression in the calibrator sample (cDNA from osteoblast cultures) and normalized to the mRNA quantity for glyceraldehyde 3-phosphate dehydrogenase (*GAPDH) *(endogenous control).

The expression of pro-inflammatory *CCL2 *was significantly higher in synovia-derived immature osteoblasts from patients with pJIA in comparison those with oJIA (*P *= 0.04, Mann-Whitney test; Figure 5). The expression of *CCL3 *(macrophage inhibitory protein (MIP)1α) was significantly higher in mature SF-derived osteoblasts from patients with pJIA than those with oJIA (*P *= 0.03, Mann-Whitney test; Figure 5). *Fas *was significantly increased in mature SF-derived osteoblasts in patients with pJIA compared with oJIA (*P *= 0.03, Mann-Whitney test; Figure 5). Expression of *TNF-α*, *IL-4*, *IL-6*, *IL-17 *and *IL-18 *was not detected in SF-derived osteoblasts, and expression of *CCL4*, *IL-1β *and *FasL *was not different between the groups.

### Effect of synovial fluid from patients with JIA on differentiation of human bone marrow-derived osteoblasts

**Figure 6:**
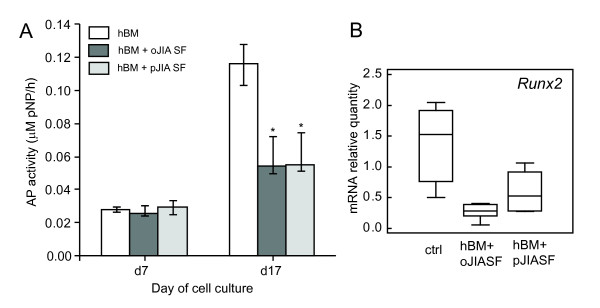
**Effect of synovial fluid from patients with oligoarticular form of juvenile idiopathic arthritis (oJIA) and polyarticular form of JIA (pJIA) on the differentiation of osteoblasts from normal human bone marrow (BM) progenitors**. Osteoblastogenesis was induced by ascorbic acid and β-glycerophosphate. Synovial fluid from patients with oJIA or pJIA was added to osteoblastogenic culture with each medium exchange. Osteoblastogenesis was assessed by AP activity and expression of *Runx2 *gene by real time PCR. **(A) **AP activity measured at day 7 and 17 in control cultures (hBM) and cultures supplemented with 10% synovial fluid from children with oJIA (hBM + oJIA SF) and pJIA (hBM + oJIA SF, * *P *< 0.05 vs. control group, Mann-Whitney test). **(B) **Expression of *Runx2 *gene in control cultures (hBM) and cultures supplemented with synovial fluid from children with oJIA (hBM + oJIA SF) and pJIA (hBM + pJIA SF). mRNA expression was calculated according to the standard curve for each gene expression in the calibrator sample (cDNA from osteoblastogenic cultures) and normalized to the mRNA quantity for glyceraldehyde 3-phosphate dehydrogenase (*GAPDH) *(endogenous control). Results are expressed as median and interquartile range (IQR) for each group; lines represent minimum and maximum values. AP, alkaline phosphatase; hBM, human bone marrow.

To assess whether soluble factors from SF of patients with JIA could inhibit differentiation of osteoblasts, we treated BM-derived osteoblasts obtained from a healthy donor with 10% of SF obtained from patients with JIA, and compared osteoblast differentiation with untreated BM osteoblast culture. Assessed by AP activity assay, SF from both oJIA and pJIA patients inhibited the differentiation of hBM-derived osteoblasts (*P *< 0.01, Kruskal-Wallis test; Figure 6A). The specificity of this effect was confirmed by inhibited expression of *Runx2 *in early osteoblastogenic culture (*P *= 0.04, Kruskal-Wallis test; Figure 6B).

## Discussion

The results of our study clearly demonstrate that osteoblastogenesis from SF-derived progenitors was impaired in patients with pJIA in comparison to patients with oJIA. Osteoblastogenesis from SF-derived progenitors correlated with systemic and local inflammatory indicators, suggesting the impact of inflammation on bone formation.

Decreased osteoblast differentiation was confirmed by the decreased area of AP-positive osteoblast colonies in SF-derived osteoblastogenic cultures from JIA patients, as well as the decreased expression of Runx2, a transcription factor essential for the commitment of mesenchymal progenitors to the osteoblast lineage [23]. We also assessed gene expression levels of *RANKL *and *OPG*, associated with osteoblast maturation and, at the same time, important for the regulation of osteoclastic bone resorption [27,28]. Decreased expression of *OPG *in SF-derived osteoblasts from patients with pJIA, together with comparable expression of *RANKL *in both patient groups, resulted in the lower *OPG/RANKL *ratio in children with pJIA, which might contribute to the increase in osteoclastic bone resorption. On the other hand, expression of *RANKL *was higher in total SF-derived cells from patients with pJIA compared to those with oJIA, which may be explained by the fact that RANKL is produced not only by osteoblasts but also by activated T lymphocytes [29]. Furthermore, both oJIA and pJIA patients expressed less *OPG *in PBMCs than the control group, which is consistent with the recent prospective cohort study reporting lower OPG serum levels, higher levels of RANKL and decreased OPG/RANKL ratio in children with JIA compared to healthy children [30].

Since oJIA had lower laboratory inflammation markers than pJIA, we expected that osteoblastogenesis would be negatively regulated by inflammatory processes. This was confirmed by the negative correlation of osteoblastogenesis with the levels of CRP and ESR, demonstrating an osteoblast-related mechanism of bone loss which accompanies autoimmune disorders [31].

Negative correlation of osteoblastogenesis with synovial IL-17 levels found in patients with JIA is consistent with IL-17 contribution to the cartilage and bone damage seen in the animal model of autoimmune arthritis [24]. IL-17 participates in the arthritic process by affecting B- and T-lymphocytes, epithelial, myelomonocytic and BM stromal cells and synovial fibroblasts and chondrocytes, stimulating their production of various cytokines, chemokines and tissue destructive mediators [32,33]. Soluble IL-17 and high numbers of IL-17-producing Th17 cells have been found in synovial tissue from adults and children with inflammatory arthritis, particularly in those with a more severe clinical course [33,34]. Although we were unable to confirm statistically significant correlation between synovial TNF-α concentration and SF-derived osteoblasts differentiation, we observed an inverse relationship between these two variables. The lack of significance for this correlation is probably related to high variability of synovial TNF-α concentration in study patients (13.03 ± 11.08 pg/ml).

In addition, we assessed whether osteoblastogenesis could be altered by therapy, but we were unable to detect a significant difference either in osteoblastogenesis or in systemic (SE, CRP) and local (synovial TNF-α) inflammation parameters between patients receiving therapy and patients without therapy. This could be ascribed to the poor therapeutic response in treated patients or activation of disease in the untreated patient group. A more homogenous and larger group of patients according to the duration of the disease and the applied therapy is needed to address this issue with adequate power.

Mesenchymal cells are known to have immunoregulatory properties [35]. Upon inflammatory stimuli, osteoblasts also may express various cytokines and chemokines that augment or suppress inflammation. Synovial inflammation in patients with RA and human TNF transgenic mice upregulates WNT5A in synovial fibroblasts [26]. WNT5A is able to induce IL-1β, IL-6, CCL2, CCL5, CXCL1, and CXCL5 in osteoblasts, thus altering their regulatory properties [26]. It has been previously shown that osteoblasts and osteocytes from RA patients immunohistochemically expressed CCL20, which was absent in osteoblasts from osteoarthritic patients [36]. We demonstrated that the expression of *CCL2 *was higher in SF-derived immature osteoblasts from patients with pJIA, whereas *CCL3 *expression was increased in mature SF-derived osteoblasts derived from patients with pJIA. In the animal model of adjuvant arthritis, CCL2 and CCL3, up-regulated by TNF and IL-1β, are shown to contribute to the pathogenesis of inflammatory arthritis [37]. Our finding of increased proinflammatory *CCL2 *and *CCL3 *in osteoblasts derived from pJIA patients suggests that osteoblastic cells in severe forms of JIA may themselves perpetuate joint inflammation via cytokine secretion. In addition, the expression of *Fas*, a TNF superfamily member characteristic for T lymphocytes but also expressed on osteoblasts and osteoclast lineage cells [38], was increased in mature SF-derived osteoblasts from pJIA patients. We have previously described that Fas expressed by mature murine osteoblasts is able to specifically inhibit their differentiation [15]. This mechanism could also be effective in human JIA and lead to suppressed osteoblasts differentiation as a result of inflammatory process in JIA.

Our study also provides experimental evidence that SF from patients with both oJIA and pJIA may adversely influence the differentiation of hBM derived osteoblasts. Together with the *in vitro *finding of Caparbo, *et al*. [39] that serum from patients with active pJIA decreases differentiation and increases apoptosis in human osteoblasts, our study experimentally demonstrated that bone loss in JIA was associated with decreased osteoblastogenesis and results in impaired bone formation. This may be the cellular mechanism for lower levels of bone formation biochemical markers found in JIA patients [40,41].

## Conclusions

In conclusion, decreased osteoblast differentiation is accompanied by altered properties of SF-derived osteoblasts from patients with severe forms of JIA, which may promote osteoclastic bone resorption and perpetuate local and systemic inflammation. Development of therapeutic interventions targeting synovial mesenchymal and/or osteoblast lineage cells in JIA would contribute to alleviating both bone destruction and inflammation in severe forms of the disease.

## Abbreviations

AP: alkaline phosphatase; CCL: CC chemokine ligand; cDNA: complementary DNA; CI: confidence interval; CRP: C-reactive protein; DMARD: disease modifying anti-rheumatic drug; ELISA: enzyme-linked immunosorbent assay; ESR: erythrocyte sedimentation rate; FasL: Fas ligand; FBS: fetal bovine serum; GAPDH: glyceraldehyde 3-phosphate dehydrogenase; Hb: haemoglobin; hBM: human bone marrow; IL: interleukin; ILAR: International League of Associations for Rheumatology; IQR: interquartile range; JIA: juvenile idiopathic arthritis; MB: methylene-blue; MIP: macrophage inhibitory protein; MSC: mesenchymal stem cells; MTX: methotrexate; NF-κB: nuclear factor κB; NSAID: non-steroid anti-inflammatory drugs; oJIA: oligoarticular form of juvenile idiopathic arthritis; OPG: osteoprotegerin; P4: fourth passage; PBMC: peripheral blood mononuclear cell; PBS: phosphate-buffered saline; PCR: polymerase chain reaction; pJIA: polyarticular form of juvenile idiopathic arthritis; pNP: p-nitrophenyl phosphate; RA: rheumatoid arthritis; RANKL: receptor activator of nuclear factor κB ligand; ρ: Spearman's coefficient rho; Runx2: runt-related transcription factor 2; SF: synovial fluid; SD: standard deviation; TNF: tumor necrosis factor.

## Competing interests

The authors declare that they have no competing interests.

## Authors' contributions

EL participated in study design, collected samples, carried out the experiments, collected data and drafted the manuscript. MJ collected blood and synovial fluid samples, collected and analyzed clinical data and helped in finalizing the manuscript. DG participated in initial planning of the study, its design and coordination, statistical analysis, and critically revised the manuscript. AM participated in study design and data analysis and critically revised the manuscript. NK designed the study, participated in experiments, performed statistical analysis, interpreted results and revised the manuscript. All authors have read and approved the final version of the manuscript.
